# Association of plant-based diet with cardiometabolic multimorbidity trajectory: A prospective study in UK Biobank

**DOI:** 10.1016/j.jnha.2026.100860

**Published:** 2026-04-25

**Authors:** Shimin Chen, Chao Chen, Li Lin, Jiayuan Wu, Zhirong Zeng

**Affiliations:** a School of Public Health, Guangdong Medical University, Dongguan, 523808 Guangdong, China; b Clinical Research Service Center, Affiliated Hospital of Guangdong Medical University, Zhanjiang, 524001 Guangdong, China; c The Affiliated Dongguan Songshan Lake Central Hospital, Guangdong Medical University, Dongguan, 523808 Guangdong, China; d Medical School of Shaoguan University, Shaoguan, 512005 Guangdong, China

**Keywords:** Plant-based diet, Cardiometabolic, Multimorbidity trajectory, Multi-state model

## Abstract

**Background:**

Plant-based diets have been consistently associated with a lower risk of several individual cardiometabolic diseases (CMDs). However, whether such dietary patterns differentially influence the progression from health to first-occurrence cardiometabolic disease (FCMD), cardiometabolic multimorbidity (CMM), and ultimately mortality remains unclear.

**Methods:**

The present study analyzed data from 83,610 participants in the UK Biobank cohort who were not diagnosed with diabetes, ischaemic heart disease (IHD), or stroke at baseline. Multi-state models were employed to examine the impact of plant-based diets on trajectories of cardiometabolic multimorbidity.

**Results:**

During a median follow-up period of 15.61 years, the median age of the participants at baseline was 57 years (IQR: 50 years-62 years), 42.72% were male. 9298 participants developed at least one CMD, 1,045 participants progressed to CMM, and 4169 participants ultimately died. The finding of the multi-state model suggest that, compared with Q1, both the overall plant-based diet index (PDI)[HR (95%CI): 0.88 (0.83, 0.94) for baseline to FCMD, 0.85 (0.83, 0.94) for baseline to CMM] and the healthy plant-based diet index (hPDI) [HR (95%CI): 0.60 (0.41, 0.89) for baseline to FCMD, 0.79 (0.53, 1.17) for baseline to CMM] were negatively associated with the risk of transitioning from health to FCMD and CMM. When grouping FCMD into disease-specific analyses, it was found that the three plant-based indices also exerted differential effects on the transition from health to diabetes.

**Conclusion:**

In the progression of CMM, high adherence of PDI and hPDI has been demonstrated to reduce the risk of transitioning from CMD-free to FCMD, particularly in diabetes, and lowers the risk of CMM with a much lower incidence risk from CMD-free to CMM compared to CMD-free to FCMD. The present study hypothesizes that both hPDI and unhealthy plant-based index (uPDI) are associated with the risk from baseline to death.

## 1 Introduction

Cardiometabolic diseases (CMDs) comprise diabetes, stroke, heart disease and other conditions, whilst cardiometabolic multimorbidity (CMM) denotes the concurrent presence of at least two CMDs [[1], [2], [3]]. These conditions are often interrelated, with the occurrence of one elevating the risk of others, posing a significant threat to middle-aged and elderly populations [4]. In 2023, approximately 440 million adults older than 55 years suffered from cardiovascular disease (CVD) globally, resulting in over 61 million disability-adjusted life years (DALYs), while the prevalence of diabetes remained persistently high [5].

As the prevalence of CMM continues to rise, its prevention and treatment have become a prominent public health issue globally [6,7]. Understanding CMM trajectories enables earlier intervention and more precise management. However, current research has insufficiently examined lifestyle determinants of CMM. Several epidemiological studies indicate that diet, as a modifiable lifestyle factor, is an effective strategy for the “three early” prevention of CMD [8,9]. High-quality diets can reduce cardiometabolic risks and mortality in middle-aged and older adults. However, current research on dietary patterns in relation to CMD and CMM remains limited. Among various dietary patterns, plant-based diets have been recognized as potentially beneficial for metabolic health due to high intake of plant-based foods and reduced intake of animal-based food [10,11]. Satija et al. proposed a plant-based diet scoring system. This system comprises the overall Plant-Based Diet Index (PDI), the Healthy Plant-Based Diet Index (hPDI), and the Unhealthy Plant-Based Diet Index (uPDI) to quantify diet quality and composition [11]. Despite evidence suggesting a potential protective effects of plant-based diets on CMD, the current body of evidence remains inadequate. The majority of research focused on examining the association between plant-based diet indices (PDIs) and a single CMD state, with limited research on CMM [10,12,13]. However, CMDs share substantial interactions and common pathophysiological mechanisms. Studying single diseases in isolation fails to capture the true burden of multimorbidity. Recent multimorbidity research has repeatedly emphasized that multimorbidity has become the norm in aging societies. The relationship between disease and diet should be studied from the perspective of integrated management and systemic interventions, rather than being confined to a single-disease model [14,15].

Previous research has examined plant-based dietary patterns in relation to the multimorbidity of cancer and cardiometabolic diseases within the UK Biobank [16]. However, that study focused on multimorbidity of cancer and CMD, without specifically investigating the trajectories of CMM. Research on the progression of CMM remains relatively limited. To address this gap, the present study, based on data from 83,610 UK Biobank participants, is the first to apply a multi-state model to examine the associations between plant-based dietary patterns and CMM trajectories. This analytical framework allows for a dynamic assessment of the associations between plant-based diets and the onset, progression, and clustering of CMD, thereby providing a more comprehensive understanding of the potential protective role of plant-based diets. In line with previous studies, we define CMD as diabetes, ischemic heart disease (IHD), and stroke [2,3].

## 2 Methods

### 2.1 Study design and participants

The UK Biobank is a large-scale prospective cohort study and biomedical database initiated by the UK government. The cohort comprises 22 research centers across the UK, recruiting over 500,000 participants. Participants were enrolled from 2006 to 2010, and a comprehensive array of data was collected via standardized sociodemographic questionnaires, obtain detailed information on participants' demographics, health factors, family history, and lifestyle. The collection process was supplemented by physical measurements, providing anthropometric data. Furthermore, comprehensive medical records were linked from the UK healthcare system, including various examination reports (e.g., imaging reports, blood tests), exposure factors (e.g., medication history, occupational health), and health-related information such as hospital admissions, discharges, cancer diagnoses, and deaths. The UK Biobank study was approved by the North West Multi-Centre Research Ethics Committee (Reference No.: 11/NW/0382), with all participants providing written informed consent.

**Fig. 1:**
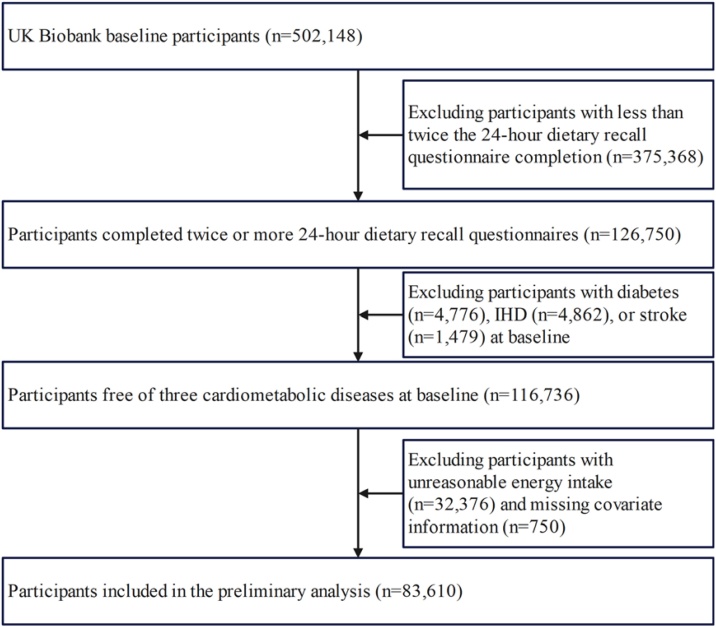
The flowchart for participant inclusion and exclusion.

This analysis utilized cohort data from 2006 to 2024, encompassing information about participants' diets, the prevalence of cardiometabolic diseases, sociodemographic details, and physical health status. After applying the inclusion and exclusion criteria shown in Fig. 1, we excluded participants who completed dietary recall questionnaires less than twice (*n* = 375,368), participants with baseline diabetes (*n* = 4776), IHD (*n* = 4862), stroke (*n* = 1479), participants with unreasonable energy intake (<800 or >4200 kcal/day in males and <600 or >3500 kcal/day in females) [17,18] (*n* = 32,376), and those with missing covariate data (*n* = 750). Ultimately, a total of 83,610 participants were included.

### 2.2 Assessment of plant-based diet score

Dietary intake was assessed using repeated 24-h dietary recalls. For participants with multiple recalls, the average intake across all available recalls was used to estimate food consumption and construct the plant-based diet indices [10,18]. This approach has been widely applied in large epidemiological studies, where repeated dietary assessments are averaged to better estimate habitual intake and reduce measurement error, although it does not fully account for intra-individual variability [19,20].

A healthful plant-based diet emphasizes consumption of whole grains, fruits, vegetables, nuts, legumes, and vegetable oils, while limiting less healthy plant foods such as refined grains, sugar-sweetened beverages and all animal foods. In contrast, an unhealthful plant-based diet prioritizes less healthy plant foods and may not provide the same cardiometabolic benefits.

The PDIs in this study consisted of 17 food groups (whole grains, fruits, vegetables, nuts, legumes, tea and coffee, refined grains, potatoes, sugar-sweetened beverages, fruit juices, sweets and desserts, animal fats, dairy products, eggs, fish or seafood, meat, and other animal foods), that were grouped into larger sub-categories: healthy plant foods, unhealthy plant foods, and animal foods. Healthy plant foods included whole grains, fruits, vegetables, nuts, legumes, and tea or coffee, which are generally minimally processed and have been consistently associated with favorable cardiometabolic health outcomes. Less healthy plant foods included refined grains, potatoes, sugar-sweetened beverages, fruit juices, and sweets or desserts, which are typically more processed and higher in refined carbohydrates or added sugars. Animal foods included animal fats, dairy products, eggs, fish or seafood, meat, and other animal foods [10,18].

The intake for each food group was divided into quintiles and assigned positive scores (Q1 to Q5 were given scores 1 to 5) or negative scores (Q1 to Q5 were given scores 5 to 1). For PDI, the plant-based food groups, including both healthy and unhealthy options, received positive scores, while all animal food groups were treated equally and assigned negative scores following the standard PDI scoring framework. To calculate hPDI, healthy plant-based food groups receive positive scores, while negative scores are assigned to unhealthy plant-based food groups and animal-based food groups. In contrast, the scoring direction for uPDI is antithetical to that of hPDI. The final scores derived from the plant-based diet are the result of a cumulative calculation of scores from 17 distinct food groups, with a theoretical range of 17 to 85 (Table S1).

### 2.3 Outcomes

For the current study, CMM is defined as having at least two the following conditions: diabetes, IHD, or stroke. Events and their occurrence times were determined through various healthcare institutions, national death registries, and self-reports during follow-up visits. All events were coded according to the International Classification of Diseases (ICD). For diabetes, the International Classification of Diseases, Tenth Revision (ICD-10) codes used were E11 and E14; for IHD, the codes were I20 to I25; and for stroke, the codes were I60 to I69 [2,3].

Follow-up continued until participant death, loss to follow-up, or September 15, 2024, whichever occurred first. All models were adjusted for age, sex, education level, the Townsend Deprivation Index, sleep quality, smoking and alcohol consumption, physical activity and family history of CMD, as well as duration of status as the time scale. For participants who entered different states on the same day, we calculated the theoretical entry date of the preceding state by subtracting 0.5 days from the entry date of the subsequent state. For example, for participants who entered CMM and death on the same day, the CMM occurrence date was the date of death minus 0.5 days [2,3].

### 2.4 Covariates

A range of factors associated with CMD status were considered as covariates: age, sex, education level, the Townsend Deprivation Index, smoking status, drinking status, sleep quality, physical activity, and family history of CMD. The Townsend Deprivation Index is a measure of socioeconomic status, calculated as a composite score based on unemployment, overcrowded housing, lack of car ownership, and lack of home ownership. A lower Townsend Deprivation Index score indicates higher socioeconomic status [21]. Alcohol consumption frequency was categorized into never drinkers, special occasions only, 1–3 times monthly, 1–2 times weekly, 3–4 times weekly, or daily/almost daily. Self-reported smoking status was classified into never smokers, former smokers, and current smokers. Sleep quality was assessed using variables including sleep duration, morning/evening person, insomnia, snoring, and daytime sleepiness/dozing [22,23]. A family history of CMD was defined as having a parent, sibling, adoptive parent, or adoptive sibling with heart disease, stroke, or diabetes. Physical activity was measured by the number of days per week that participants engaged in at least 10 min of moderate- or vigorous-intensity exercise (Table S2).

### 2.5 Statistical analysis

In the initial analyses, Quantitative data following a normal distribution are presented as the mean ± standard deviation, with intergroup comparisons conducted using independent samples t-tests or one-way ANOVA. For quantitative data that does not meet normality assumption, the median (interquartile range) was reported, with intergroup comparisons performed using Mann-Whitney *U* test or Kruskal-Wallis H rank sum test. Qualitative data were presented as case numbers (%), with intergroup comparisons conducted using chi-square tests or Fisher's exact tests. Cox proportional hazards regression models were used to estimate the association between different types of PDIs and FCMD, CMM, and all-cause mortality, with these dietary scores incorporated as categorical variables. After Cox regression, multistate models were employed to explore the contribution of PDIs during transitions between CMD-free, FCMD, CMM, and death, with death incorporated as an absorbing state in the transition model. Similarly, PDIs were incorporated into the multi-state model in quartiles, with each quartile being treated as an ordinal variable to capture trends across categories. Multi-state models are an extension of competing risks models and Markov chains. The distinguishing feature of multi-state models is their ability to simultaneously describe the influence of risk factors on different stages of disease progression while also taking competing risks into account [24]. In accordance with preceding studies [[1], [2], [3],^25^], a six-stage model of the CMM development pathway was constructed (Transition Pattern A, Figure S1): 1) CMD-free to FCMD; 2) CMD-free to CMM; 3) FCMD to CMM; 4) CMD-free to death; 5) FCMD to death; 6) CMM to death. Furthermore, to comprehensively analyze the differential roles of distinct FCMD types in CMM progression, new pathways were reconfigured based on FCMD subtypes (diabetes, IHD, and stroke), forming 12 transition phases (Transition Pattern B, Figure S2).

Furthermore, stratified analyses were conducted to evaluate potential heterogeneity in the association between PDIs and CMM trajectory across subgroups defined by age, sex, body mass index (BMI), and family history of CMD. BMI was calculated as weight in kilograms divided by height in meters squared (kg/m²), with height and weight measured by trained personnel following standardized protocols.

All analyses were performed on R (version 4.1.3), and the multi-state models were run using the R "mstate" package. A two-tailed p-value < 0.05 was considered statistically significant.

## 3 Results

### 3.1 Descriptive analysis

**Fig. 2:**
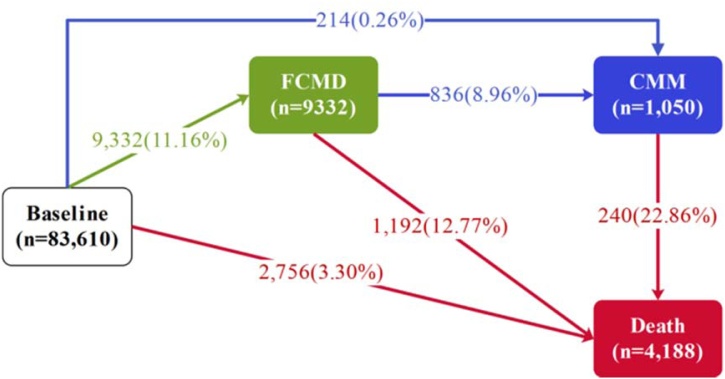
Numbers (percentages) of participants in transition pattern A.

**Fig. 3:**
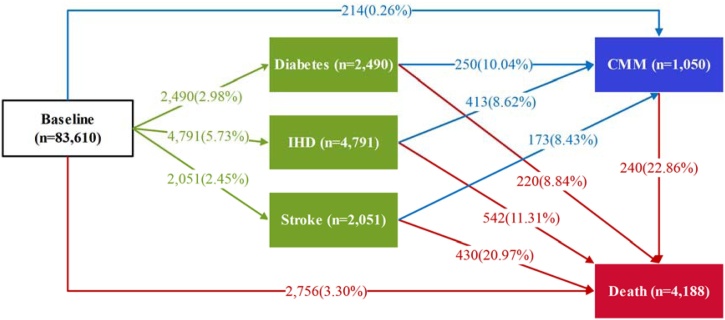
Numbers (percentages) of participants in transition pattern B.

The median age of the participants at baseline was 57 years (IQR: 50-62 years), 42.72% were male. The median values for overall PDI, hPDI, and uPDI were 51 (IQR: 48, 55), 51 (IQR: 46, 55), and 50 (IQR: 46, 55), respectively (Table S3). With a median follow-up of 15.61 years (IQR: 14.95–16.23 years; total person-years (PYs) 1,282,546). Among 83,610 participants, 214 progressed directly from CMD-free to CMM, 9332 (11.16%) had at least one CMD of whom 836 (8.96%) further progressed to CMM. During follow-up, 4188 participants died, including 1192 in the FCMD state and 240 in the CMM state (Fig. 2). When FCMD was further categorized into specific CMDs, 2490 participants (2.98%) had diabetes, 4791 (5.73%) had IHD, and 2051 (2.45%) had stroke (Fig. 3). Compared with participants without CMM during follow-up, those with CMM were more likely to be male, older, have higher BMI, lower socioeconomic status, previous smoker, higher frequency of drinking, have family history of CMD, lower hPDI and higher uPDI (Table S4).

### 3.2 Cox regression analyses

The results of Cox proportional hazards models examining the quartiles of three different plant-based dietary scores (PDI, hPDI, and uPDI) against risks of FCMD, CMM, and all-cause mortality. PDI and hPDI showed significant negative correlations with FCMD, CMM, and all-cause mortality, while uPDI exhibited a significant positive correlation. After adjusting for all covariates, both FCMD risk and all-cause mortality showed a marked downward trend with increasing PDI adherence. When the adherence to PDI reached the highest level, the corresponding HRs (95% CIs) were 0.88 (0.83, 0.94) and 0.86 (0.79, 0.94), respectively. For the outcome of CMM, the HRs for PDI Q2 and Q3 were close (0.84 vs 0.85), while Q4 showed a dramatic decrease (HR: 0.69). To investigate the underlying reasons, a restrictive cubic spline model using the proportion of healthy plant-based foods in the PDI as a continuous variable. CMM risk declined rapidly at low proportions, plateaued at moderate levels, and then decreased further before stabilizing at high proportions (Figure S3). Compared to participants in the lowest hPDI quartile (Q1), higher hPDI quartiles (Q2–Q4) were associated with lower mortality risk, though a slight upward trend in Q4. When the proportion of healthy plant-based foods in hPDI was modeled continuously in a restricted cubic spline, mortality risk decreased significantly with increasing proportion of healthy plant-based foods, reaching a minimum around 30–40% before the curve flattened (Figure S4). The quartiles of uPDI showed a clear upward trend in risk for FCMD [Q4 vs Q1: HR (95% CI): 1.15 (1.08, 1.22)], CMM [Q4 vs Q1: HR (95% CI): 1.28 (1.06, 1.53)], and mortality [Q4 vs Q1: HR (95% CI): 1.23 (1.12, 1.35)] (Table 1).

**Table 1:** Associations of incident FCMD, CMM, and all-cause mortality by PDIs among 83,610 participants.

		Q1	Q2	Q3	Q4	*P* for trend
PDI^a^	FCMD	Ref	**0.94 (0.89, 0.99)**	**0.93 (0.88, 0.98)**	**0.88 (0.83, 0.94)**	<0.001
	CMM	Ref	**0.84 (0.72, 0.99)**	0.85 (0.72, 1.01)	**0.69 (0.57, 0.83)**	<0.001
	Death	Ref	**0.92 (0.84, 0.99)**	**0.88 (0.81, 0.96)**	**0.86 (0.79, 0.94)**	<0.001
hPDI^b^	FCMD	Ref	**0.90 (0.85, 0.94)**	**0.89 (0.84, 0.94)**	**0.85 (0.80, 0.90)**	<0.001
	CMM	Ref	0.87 (0.74, 1.01)	**0.80 (0.68, 0.95)**	**0.78 (0.65, 0.94)**	0.003
	Death	Ref	0.92 (0.85, 1.00)	**0.86 (0.79, 0.93)**	**0.90 (0.82, 0.98)**	0.003
uPDI^c^	FCMD	Ref	1.04 (0.99, 1.10)	1.05 (0.99, 1.11)	**1.15 (1.08, 1.22)**	<0.001
	CMM	Ref	1.08 (0.91, 1.27)	**1.23 (1.04, 1.46)**	**1.28 (1.06, 1.53)**	0.003
	Death	Ref	**1.08 (1.00, 1.17)**	**1.09 (1.00, 1.19)**	**1.23 (1.12, 1.35)**	<0.001

Bold text indicates that the association between the level of the exposure factor and the outcome is statistically significant (i.e., P < 0.05).

a PDI, plant-based diet index.

b hPDI, healthy plant-based diet index.

c uPDI, unhealthy plant-based diet index.

### 3.3 Multi-state analyses

The distinct roles of PDIs in each transition stage of CMD was assessed by using multi-state models. Higher PDI quartiles were associated with a reduced risk of FCMD occurrence. Compared with the lowest quartile (Q1), the HR and 95% CIs for PDI Q4 was 0.88 (0.83, 0.94). Exposure to different PDIs showed varying associations with disease progression from baseline to diabetes. Higher PDI was significantly associated with a reduced risk of progression from baseline to diabetes [Q4 vs Q1: HR (95% CI): 0.85 (0.76, 0.96)], but was not associated with IHD or stroke. Similarly, hPDI showed a pronounced negative correlation with diabetes occurrence, with the highest quartile exhibiting particularly marked risk reduction [Q4 vs Q1: HR (95%CI): 0.64 (0.57, 0.73)]. Conversely, uPDI was positively associated with diabetes risk [Q4 vs Q1: HR (95%CI): 1.23 (1.10, 1.39)].

Regarding progression from baseline to CMM, both PDI and hPDI demonstrated the effect of risk reduction, with the protective effect of PDI Q4 being the most pronounced compared to Q1 [HR (95%CI): 0.62 (0.42, 0.91)]. hPDI showed a similar trend, but with weaker consistency across quantiles. No significant association was found between uPDI and CMM progression.

Furthermore, higher hPDI was associated with reduced risk of progression from CMD-free to all-cause mortality, most notably in hPDI Q4 [HR (95%CI): 0.88 (0.78, 0.98)]. In contrast, the highest uPDI quartile was associated with increased baseline→mortality risk [HR (95%CI): 1.26 (1.12, 1.41)]. Transitions from specific FCMD statuses to CMM or death showed no significant association with most PDIs (Tables 2, S5).

**Table 2:** Associations of PDIs (in quartiles) with trajectory of CMM.

	PDI^a^			hPDI^b^			uPDI^c^		
Transitions	Q2 vs Q1	Q3 vs Q1	Q4 vs Q1	Q2 vs Q1	Q3 vs Q1	Q4 vs Q1	Q2 vs Q1	Q3 vs Q1	Q4 vs Q1
Baseline → FCMD	**0.94 (0.89, 0.99)**	**0.93 (0.88, 0.98)**	**0.88 (0.83, 0.94)**	**0.90 (0.85, 0.94)**	**0.89 (0.84, 0.94)**	**0.85 (0.80, 0.90)**	1.04 (0.99, 1.10)	1.05 (0.99, 1.11)	**1.15 (1.08, 1.22)**
Baseline → CMM	**0.59 (0.41, 0.85)**	**0.68 (0.48, 0.97)**	**0.62 (0.42, 0.91)**	0.78 (0.55, 1.10)	**0.67 (0.46, 0.98)**	0.78 (0.52, 1.16)	0.87 (0.59, 1.28)	**1.43 (1.00, 2.04)**	1.19 (0.79, 1.80)
FCMD → CMM	0.96 (0.80, 1.15)	0.94 (0.78, 1.13)	**0.78 (0.64, 0.97)**	0.98 (0.83, 1.17)	0.89 (0.73, 1.07)	0.88 (0.72, 1.09)	1.09 (0.91, 1.31)	1.15 (0.95, 1.39)	1.17 (0.95, 1.44)
Baseline → Death	0.93 (0.84, 1.03)	0.92 (0.82, 1.02)	0.90 (0.81, 1.01)	0.91 (0.83, 1.01)	**0.86 (0.78, 0.96)**	**0.88 (0.78, 0.98)**	**1.13 (1.02, 1.25)**	1.10 (0.99, 1.22)	**1.26 (1.12, 1.41)**
FCMD → Death	0.91 (0.78, 1.05)	**0.82 (0.70, 0.96)**	0.90 (0.77, 1.06)	1.06 (0.91, 1.23)	0.93 (0.79, 1.09)	1.06 (0.90, 1.26)	0.95 (0.82, 1.11)	1.04 (0.89, 1.22)	1.01 (0.85, 1.20)
CMM → Death	1.28 (0.92, 1.77)	1.13 (0.80, 1.59)	0.81 (0.53, 1.26)	0.85 (0.61, 1.18)	0.89 (0.63, 1.28)	1.15 (0.79, 1.67)	0.86 (0.60, 1.22)	0.95 (0.66, 1.36)	1.26 (0.87, 1.83)

Bold text indicates that the association between the level of the exposure factor and the outcome is statistically significant (i.e., P < 0.05).

a PDI, plant-based diet index.

b hPDI, healthy plant-based diet index.

c uPDI, unhealthy plant-based diet index.

### 3.4 Stratified analysis

Stratified analysis revealed that the association between PDI and each subgroup was consistent overall. Regarding progression from FCMD to CMM, PDI showed a stronger negative correlation among participants with BMI < 25 kg/m^2^[HR (95% CI): 0.76 (0.62, 0.93)], with significant interaction of BMI (*P_interaction_* = 0.014). The transition from CMM to death revealed a significant sex interaction (*P_interaction_* <0.001): a negative correlation was observed in female [HR (95%CI): 0.51 (0.35, 0.73)], whereas no such association was found in male. After stratification by age, hPDI showed significant interactions with baseline to FCMD, FMCD to CMM, and CMM to death, while uPDI exhibited similar associations with baseline to FCMD (Figures S5–S7).

## 4 Discussion

The present study investigated the impact of PDIs on the onset and progression trajectories of CMD from the UK Biobank cohort. Results demonstrated that higher adherence to PDI and hPDI was associated with a reduced risk of multiple transitions, from baseline to FCMD and baseline to CMM. Additionally, hPDI and uPDI exerted effects from baseline to mortality. Notably, when FCMD was subdivided into specific CMDs, three PDIs were observed to exert distinct effects only on the transition from baseline to diabetes.

Although Córdova et al. reported protective associations between healthful plant-based diets and the multimorbidity of cancer and CMD, their analysis focused on combined cancer–CMD outcomes, whereas our study specifically modeled CMD trajectories [16]. By delineating sequential transitions from CMD-free status to first-onset CMD and subsequent multimorbidity, our findings extend previous evidence and underscore the potential of plant-based dietary patterns for both primary and secondary prevention of cardiometabolic multimorbidity.

Several biological mechanisms may underlie the protective role of healthful plant-based diets in cardiometabolic diseases. A key feature of healthful plant-based diets is their high content of dietary fiber, which may improve glycemic control by slowing glucose absorption and enhancing insulin sensitivity [26]. At the same time, these diets are typically rich in polyphenols and other antioxidant compounds that help attenuate oxidative stress and low-grade inflammation, both of which are central to the development of cardiometabolic diseases [27]. In addition, the fat profile of plant-based diets differs substantially from that of animal-based diets, with a greater proportion of unsaturated fatty acids from sources such as nuts and vegetable oils. This shift has been consistently linked to more favorable lipid profiles [28]. There is also growing evidence that plant-based dietary patterns can influence gut microbiota composition, which in turn may affect metabolic regulation and inflammatory pathways [29]. Importantly, these potential benefits are not only driven by what is increased, but also by what is reduced. Lower consumption of animal-derived foods may decrease exposure to saturated fats and other components associated with insulin resistance and endothelial dysfunction. Taken together, these pathways may jointly contribute to delaying disease onset and slowing progression toward multimorbidity.

In further disease-specific analysis of FCMD, we did not observe a significant association between PDIs and stroke. This negative finding contrasts unfavorably with multiple large prospective studies, such as those from the Nurses’ Health Study (NHS) and Health Professionals Follow-Up Study (HPFS) cohorts, which reported a significant negative correlation between hPDI and stroke, while uPDI showed a positive correlation [13]. Research indicates that there are significant differences in the pathophysiological mechanisms underlying ischemic and hemorrhagic strokes. The theory posits that plant-based diets exert a more pronounced protective effect against ischemic stroke through mechanisms such as improving lipid profiles [11]. The current study did not perform a subgroup analysis, and the limited number of stroke events (*n* = 2042) may have restricted statistical power and obscured potential associations [30,31].

A higher healthful plant-based diet index (hPDI) was significantly associated with a reduced risk of both FCMD, CMM and death, with the protective effect being markedly stronger against the progression to CMM, suggesting that greater adherence to diets rich in healthy plant foods may play an important role in preventing the onset and progression of cardiometabolic diseases. This stronger association with CMM may reflect the additive or synergistic protective effects of plant-based diets in environments of heightened biological vulnerability [32]. Following the initial onset of conditions such as hypertension, the body often enters a state of accelerated metabolic dysfunction, heightened oxidative stress, and chronic inflammation [33]. In this context, the multifaceted benefits of healthful plant-based dietary patterns, including improved insulin sensitivity, better lipid profiles, enhanced endothelial function, and reduced systemic inflammation which may prove particularly critical and efficient in preventing other diseases [34,35]. It not only prevents individual diseases but also effectively curbs the spread of dysfunction across multiple organ systems [36]. Furthermore, individuals already suffering from FCMD represent a high-risk population for CMM. In the general healthy population, the absolute risk of multimorbidity is substantially higher than that of first-onset illness. Therefore, any protective factor (such as a healthy diet) may prevent a greater number of absolute events in this high-risk cohort, making its benefits more detectable in disease-progression models. For individuals diagnosed with FCMD, lifestyle modifications toward healthier dietary habits are more likely after diagnosis. Importantly, the shift from modest effects in primary prevention to stronger effects in halting disease progression suggests that promoting plant-based diets holds substantial clinical and public health value. From a translational perspective, these findings support integrating healthful plant-based dietary guidance into secondary and tertiary prevention strategies, particularly for individuals already affected by cardiometabolic diseases. Improving the quality of plant-based diets, as reflected by a higher hPDI score, involves not only increasing the intake of healthy plant foods—such as whole grains, fruits, vegetables, nuts, and legumes—but also reducing the consumption of less healthy plant foods, including refined grains and sugar-sweetened beverages, as well as limiting animal-derived foods. Early dietary counseling and sustained adherence to such high-quality plant-based dietary patterns may help mitigate disease clustering, reduce healthcare burden, and improve long-term cardiometabolic outcomes at the population level.

Our findings reveal the significant impact of plant-based diets on cardiometabolic health, carrying major public health implications. First, this study corroborates the notion that higher quality plant-based diets, reflected by greater adherence to a healthful plant-based diet index (hPDI), are significantly associated with reduced CMD risk. Importantly, we found that plant-based diet quality not only substantially influences the transition from CMD-free to FCMD but also impacts the critical progression from CMD-free to CMM—the same-day diagnosis of two or more CMDs in CMD-free individuals—a latter association that has received relatively less research attention. Given the substantial burden of CMM in terms of disability, healthcare costs, and premature mortality, our findings suggest that public health strategies should be developed and implemented to promote healthful plant-based dietary patterns characterized by higher consumption of whole grains, fruits, vegetables, nuts, and legumes and lower intake of refined grains and sugar-sweetened beverages. The objective of these strategies is to prevent disease progression and reduce the overall burden of multiple chronic diseases. Furthermore, against the backdrop of global population aging—a demographic shift accompanied by rising chronic disease prevalence (particularly CMD [37]) and increased DALYs loss-the findings underscore the urgency of addressing these challenges. This study emphasizes that improving dietary quality represents a scalable and effective approach to promote healthy aging. Adopting a diet pattern centered on healthy plant-based foods can extend healthy life expectancy and reduce the socioeconomic burden on aging societies. Finally, the findings indicate that the benefits of a plant-based diet are particularly pronounced among high-risk groups, such as those with lower socioeconomic status or limited access to healthy foods, which is a critical opportunity for implementing targeted interventions, as improving dietary quality among vulnerable populations not only helps reduce nutrition-related disparities but also alleviates the disproportionate burden they bear. Therefore, public health initiatives to enhance access to healthy plant-based foods can serve as a powerful tool for addressing these challenges.

This study possesses several advantages. First, compared to traditional Cox proportional hazards models that examine single events, the multi-state model facilitates the elucidation of the dynamic role of PDIs in the progression of CMD. Second, the large sample size of the UK Biobank and outcome data based on objective clinical diagnoses minimize outcome classification errors, enhancing the validity of the findings. Secondly, the large sample size and prospective design based on participants from the UK Biobank provide substantial statistical power and reduce the likelihood of reverse causation. In addition, the use of plant-based diet indices enabled us to differentiate between overall plant-based diets and the quality of plant foods, providing more nuanced insights into the role of dietary patterns in cardiometabolic disease progression.

Meanwhile, several limitations of this study warrant attention. First, methodological heterogeneity exists across studies when constructing PDIs using 24 -h dietary recall data [18,38]. Differences in the classification of food categories and the allocation of scoring for plant-based versus animal-based foods may lead to inconsistent results when comparing findings, thereby limiting the direct comparability of this study's results with other researches in this field. Second, the definition of CMD has not been standardized. Different studies employ varying disease combinations (e.g., inclusion of fatty liver disease, specific diabetes subtypes, or stroke subtypes) and diagnostic criteria [25,39]. This heterogeneity in the literature complicates direct comparisons of incidence rates and risk estimates and may affect the generalizability of findings to populations or clinical settings using alternative definitions. Third, data on key lifestyle and socioeconomic covariates (e.g., physical activity, smoking status, sleep quality) were based on self-reported questionnaire responses. While widely used in large-scale epidemiological studies, these measurement methods are inherently susceptible to measurement error, recall bias, and social desirability bias, potentially introducing residual confounding. In addition, the PDI was calculated based on the mean intake from repeated 24-h dietary recalls. Although averaging multiple recalls may reduce random measurement error compared with a single recall, this approach does not fully account for within-person variability in dietary intake, which may lead to some degree of exposure misclassification.

## 5 Conclusion

The results of this study indicate that greater adherence to PDI and hPDI is associated with a reduced risk of transitioning from CMD-free to FCMD, particularly diabetes, and a lower risk of CMM. Furthermore, both hPDI and uPDI were associated with mortality risk from baseline to death. The observed protective associations may be explained by the high content of dietary fiber, antioxidants, and unsaturated fatty acids in healthful plant-based foods, which contribute to improved insulin sensitivity, lipid metabolism, and systemic inflammation. Collectively, this evidence underscores the importance of promoting adherence to PDI and hPDI for both primary and secondary prevention of CMM.

## CRediT authorship contribution statement

**S.M.C**: Conceptualization, Methodology, formal analysis, literature screening, Writing - original draft preparation. **C.C.**: Conceptualization, Methodology, visualization, Writing - reviewing & editing, formal analysis. **L.L.**: formal analysis, Writing - reviewing & editing. **J.Y.W.**: Conceptualization, Methodology, visualization, literature screening, Writing - reviewing & editing. **Z.R.Z.**: Conceptualization, methodology, Writing - reviewing & editing, supervision.

## Ethics approval and consent to participate

The UK Biobank study was approved by the North West Multi-Centre Research Ethics Committee (Reference No.: 11/NW/0382), with all participants providing written informed consent.

## Declaration of Generative AI and AI-assisted technologies in the writing process

Not applicable.

## Funding

This work was supported by the National Science and Technology Innovation 2030, Noncommunicable Chronic Diseases-National Science and Technology Major Project (Grant No.2023ZD0508500, 2023ZD0508506).

## Data statement

All data used in this study were obtained from the UK Biobank. Researchers can access the data used in this study by visiting the UK Biobank website.

## Declaration of competing interest

The authors declare that they have no competing interests.

## Footnotes

Appendix A Supplementary material related to this article can be found, in the online version, at doi:https://doi.org/10.1016/j.jnha.2026.100860.
