# Supplementary material for: Association of plant-based diet with cardiometabolic multimorbidity trajectory: A prospective study in UK Biobank
Source: J Nutr Health Aging. 2026 Apr 25;30(6):100860. doi: 10.1016/j.jnha.2026.100860 (PMC13134018; doi:10.1016/j.jnha.2026.100860)
Supplement: Supplementary file 1 [file mmc1.docx]

# **Supplementary material**

**Content**

[**Supplementary material** 1](#_Toc214720866)

[**Table S1 Food Groups and Scoring of the Plant-Based Diet Indices** 2](#_Toc214720867)

[**Table S2 The Field ID and definition of covariates** 4](#_Toc214720868)

[**Table S3 Baseline characteristics of 83,610 participants in the UK Biobank cohort by incident disease status during follow-up.** 5](#_Toc214720869)

[**Table S4 Comparison of baseline characteristics of participants who suffer with CMM to who suffer without CMD or with one CMD.** 7](#_Toc214720870)

[**Table S5 Associations of PDIs (in quartiles) with trajectory of CMM (specific FCMD).** 8](#_Toc214720871)

[**Figure S1 CMM transition pattern A** 10](#_Toc214720872)

[**Figure S2 CMM transition pattern B** 10](#_Toc214720873)

[**Figure S3 Associations of for incident CMM by proportion of healthy plant-based foods in the PDI** 11](#_Toc214720874)

[**Figure S4 Associations of for incident all-cause mortality by the proportion of healthy plant-based foods in the hPDI** 12](#_Toc214720875)

[**Figure S5 Associations of PDI with CMM transitions among 83,610 participants, stratified by potential modifiers.** 13](#_Toc214720876)

[**Figure S6 Associations of hPDI with CMM transitions among 83,610 participants, stratified by potential modifiers.** 15](#_Toc214720877)

[**Figure S7 Associations of uPDI with CMM transitions among 83,610 participants, stratified by potential modifiers.** 17](#_Toc214720878)

## **Table S1 Food Groups and Scoring of the Plant-Based Diet Indices**

| **Food groups** | **Food items** | **Scoring for PDI** | **Scoring for hPDI** | **Scoring for uPDI** |
| --- | --- | --- | --- | --- |
| **Plant-based food groups** | | | | |
| **Healthy** |  |  |  |  |
| Whole grains | Porridge, muesli, plain cereal, bran cereal, whole-wheat cereal, oatcakes, wholemeal bread (flour type: wholemeal), whole meal pasta, brown rice, couscous, other cooked grains (such as bulgur). | Positive | Positive | Reverse |
| Fruits | Stewed fruit, prune, dried fruit, mixed fruit, apple, banana, berries, cherries, grapefruit, grapes, mango, melon, orange, orange-like small fruits, peach/nectarine, pear, pineapple, plum, other fruits, olives | Positive | Positive | Reverse |
| Vegetables | Mixed vegetables, vegetable pieces, coleslaw, side salad, avocado, beetroot, broccoli, butternut squash, cabbage/kale, carrots, cauliflower, celery, courgette, cucumber, garlic, leeks, lettuce, mushrooms, onion, parsnip, sweet peppers, spinach, sprouts, sweetcorn, fresh tomatoes, cooked or tinned tomatoes, turnip/swede, watercress, other vegetable intake | Positive | Positive | Reverse |
| Nuts | Salted peanuts, unsalted peanuts, salted nuts, unsalted nuts, seeds | Positive | Positive | Reverse |
| Legumes | Vegetarian sausages/burgers, tofu, quorn, other vegetarian alternative, baked beans, pulse, broad beans, green beans, peas | Positive | Positive | Reverse |
| Tea and coffee | Instant coffee, filtered coffee, cappuccino, latte, espresso, other coffee drinks, standard tea, rooibos tea, green tea, herbal tea, other tea, low calorie hot chocolate, hot chocolate | Positive | Positive | Reverse |
| **Less healthy** |  |  |  |  |
| Refined grains | Oat crunch, sweetened cereal, other cereal, white bread (flour types: white, mix, and others), naan bread, garlic bread, crispbread, other bread, white pasta, white rice, snackpot, couscous, pancake, scotch pancake, croissant, scone, savoury biscuits, cheesy biscuits, sweet potato, other savoury snack | Positive | Reverse | Positive |
| Potatoes | Fried potatoes, boiled/baked potatoes, mashed potatoes, crisps (e.g., potato chips) | Positive | Reverse | Positive |
| Sugary drinks | Low calorie or diet drinks (e.g., fizzy, squash), carbonated (fizzy) drinks, squash or cordial | Positive | Reverse | Positive |
| Fruit juices | Orange juice, grapefruit juice, other fruit/vegetable juice, fruit smoothie | Positive | Reverse | Positive |
| Sweets and desserts | Double crust pie, single crust pie/flan, crumble topping, Yorkshire pudding, Danish pastry, fruitcake, cake, doughnuts, sponge pudding, other dessert, chocolate bar, white chocolate, milk chocolate, dark chocolate, chocolate-covered raisin, chocolate sweet, diet sweets, chocolate-covered biscuits, chocolate biscuits, sweet biscuits, other sweets | Positive | Reverse | Positive |
| **Animal-based food groups** | | | | |
| Animal fat | Butter on bread/crackers (spreadable, low fat, normal fat, or unknown type), dairy spread on bread/crackers (very low fat, low fat, normal fat, unknown type) | Reverse | Reverse | Reverse |

## **Table S2 The Field ID and definition of covariates**

| **Variables** | **Field ID** | **Unit** | **Notes** |
| --- | --- | --- | --- |
| Age | 21022 | years | The age is a derived variable calculated based on the participant's date of birth and the date of their initial assessment center visit, representing the participant's age on the day of the initial assessment center visit, rounded to the nearest full year. |
| Gender | 31 |  | Sex is acquired from central registry at recruitment, but in some cases is updated by the participant. Hence, this field may contain a mixture of the sex the NHS had recorded for the participant and the self-reported sex. |
| Education level | 6138 |  | Educational level is further categorized based on UK degree classifications into those with a university degree and those without a university degree. |
| Townsend Deprivation Index | 22189 |  | The Townsend deprivation index is calculated immediately prior to the participant joining the UK Biobank. Based on the preceding national census output areas. Each participant is assigned a score corresponding to the output area in which their postcode is located. |
| Smoking status | 20116 |  | The current/past smoking status of the participant. |
| Alcohol consumption frequency | 1558 |  | The frequency of alcohol consumption among participants |
| Sleep quality | 1160, 1180, 1200, 1210, 1220 |  | Sleep duration, chronotype, sleeplessness/insomnia, snoring, and daytime dozing/sleeping are used to calculate participants' healthy sleep scores. |
| Physical activity | 884, 904 | days | The number of days in a week that you do moderate physical activities  for at least 10 minutes continuously at a time, including activities that participants do for work, leisure, travel and around the house. |
| Family history of CMD | 20107,20110, 20111, 20112, 20113, 20114 |  | Family history of CMD is defined as a history of diabetes, stroke, and heart disease among parents, siblings, adopted parents, and adopted siblings, considering the influence of genetic and environmental factors. |

## **Table S3 Baseline characteristics of 83,610 participants in the UK Biobank cohort by incident disease status during follow-up.**

| **Characteristic** | **Total** | **CMD free** | **FCMD survivor** | **CMM survivor** | **Dead** | ***P*** |
| --- | --- | --- | --- | --- | --- | --- |
| **Age at baseline, years** | 57 (50, 62) | 56 (49, 61) | 60 (55, 64) | 61 (57, 65) | 62 (57, 66) | <0.001 |
| **Sex** |  |  |  |  |  | <0.001 |
| Male | 35,720 (42.72) | 28,712 (40.26) | 4,221 (57.79) | 513 (63.33) | 2,274 (54.30) |  |
| Female | 47,890 (57.28) | 42,596 (59.74) | 3,083 (42.21) | 297 (36.67) | 1,914 (45.70) |  |
| **Degree** |  |  |  |  |  | <0.001 |
| College or university degree | 41,129 (49.19) | 35,947 (50.41) | 3,076 (42.11) | 289 (35.68) | 1,817 (43.39) |  |
| No college or university degree | 42,481 (50.81) | 35,361 (49.59) | 4,228 (57.89) | 521 (64.32) | 2,371 (56.61) |  |
| **Townsend Deprivation Index** | -2.49 (-3.86, -0.19) | -2.50 (-3.87, -0.21) | -2.49 (-3.86, -0.16) | -2.38 (-3.73, 0.12) | -2.43 (-3.80, -0.05) | 0.038 |
| **BMI, kg*m^-2^** | 25.82 (23.45, 28.75) | 25.61 (23.29, 28.43) | 27.47 (24.77, 30.77) | 28.51 (25.71, 32.05) | 26.57 (23.94, 29.74) | <0.001 |
| **Smoking status** |  |  |  |  |  | <0.001 |
| Never smoke | 48,802 (58.37) | 42,765 (59.97) | 3,733 (51.11) | 368 (45.43) | 1,936 (46.23) |  |
| Previous smoke | 28,987 (34.67) | 23,918 (33.54) | 2,962 (40.55) | 353 (43.58) | 1,754 (41.88) |  |
| Current smoke | 5,821 (6.96) | 4,625 (6.49) | 609 (8.34) | 89 (10.99) | 498 (11.89) |  |
| **Alcohol consumption frequency** |  |  |  |  |  | <0.001 |
| Never drink alcohol | 4,569 (5.46) | 3,719 (5.22) | 477 (6.53) | 52 (6.42) | 321 (7.66) |  |
| Special occasions only | 7,412 (8.86) | 6,143 (8.61) | 764 (10.46) | 82 (10.12) | 423 (10.10) |  |
| One to three times a month | 8,919 (10.67) | 7,689 (10.78) | 764 (10.46) | 101 (12.47) | 365 (8.72) |  |
| Once or twice a week | 20,790 (24.87) | 17,864 (25.05) | 1,770 (24.23) | 193 (23.83) | 963 (22.99) |  |
| Three or four times a week | 22,037 (26.36) | 19,178 (26.89) | 1,726 (23.63) | 186 (22.96) | 947 (22.61) |  |
| Daily or almost daily | 19,883 (23.78) | 16,715 (23.44) | 1,803 (24.69) | 196 (24.20) | 1,169 (27.91) |  |
| **Sleep quality** | 3 (2, 3) | 3 (2, 3) | 3 (2, 4) | 3 (2, 3) | 3 (2, 3) | <0.001 |
| **Family history of CMD** |  |  |  |  |  | <0.001 |
| Yes | 41,214 (49.29) | 34,596 (48.52) | 4,150 (56.82) | 488 (60.25) | 1,980 (47.28) |  |
| No | 42,396 (50.71) | 36,712 (51.48) | 3,154 (43.18) | 322 (39.75) | 2,208 (52.72) |  |
| **Physical exercise, days** | 5 (2, 8) | 5 (2, 8) | 5 (2, 7) | 5 (2, 7) | 5 (2, 7) | <0.001 |
| **PDI** | 51 (48, 55) | 51 (48, 55) | 51 (47, 55) | 51 (47, 54) | 51 (47, 55) | <0.001 |
| **hPDI** | 51 (46, 55) | 51 (46, 55) | 50 (45, 55) | 49 (45, 54) | 50 (45, 55) | <0.001 |
| **uPDI** | 50 (46, 55) | 50.0 (46, 55) | 50.0 (46, 55) | 51 (46, 5) | 51 (46, 55) | 0.019 |

## **Table S4 Comparison of baseline characteristics of participants who suffer with CMM to who suffer without CMD or with one CMD.**

| **Characteristic** | **No CMD or one CMD** | **Suffer with CMM** | ***P*** |
| --- | --- | --- | --- |
| **Age at baseline, years** | 57 (50, 62) | 62 (57, 66) | <0.001 |
| **Sex** |  |  | <0.001 |
| Male | 35,032 (42.43) | 688 (65.52) |  |
| Female | 47,528 (57.57) | 362 (34.48) |  |
| **Degree** |  |  | <0.001 |
| College or university degree | 40,748 (49.36) | 381 (36.29) |  |
| No college or university degree | 41,812 (50.64) | 669 (63.71) |  |
| **Townsend Deprivation Index** | -2.50 (-3.87, -0.19) | -2.32 (-3.69, 0.31) | 0.004 |
| **BMI, kg*m^-2^** | 25.80 (23.42, 28.70) | 28.44 (25.64, 32.04) | <0.001 |
| **Smoking status** |  |  | <0.001 |
| Never smoke | 48,355 (58.57) | 447 (42.57) |  |
| Previous smoke | 28,510 (34.53) | 477 (45.43) |  |
| Current smoke | 5,695 (6.90) | 126 (12.00) |  |
| **Alcohol consumption frequency** |  |  | 0.11 |
| Never drink alcohol | 4,498 (5.45) | 71 (6.76) |  |
| Special occasions only | 7,309 (8.85) | 103 (9.81) |  |
| One to three times a month | 8,802 (10.66) | 117 (11.14) |  |
| Once or twice a week | 20,548 (24.89) | 242 (23.05) |  |
| Three or four times a week | 21,784 (26.39) | 253 (24.10) |  |
| Daily or almost daily | 19,619 (23.76) | 264 (25.14) |  |
| **Sleep quality** | 3 (2, 3) | 3 (2, 3) | 0.019 |
| **Family history of CMD** |  |  | <0.001 |
| Yes | 40,601 (49.18) | 613 (58.38) |  |
| No | 41,959 (50.82) | 437 (41.62) |  |
| **Physical exercise, days** | 5 (2, 8) | 5 (2, 7) | <0.001 |
| **PDI** | 51 (48, 55) | 51 (47, 54) | <0.001 |
| **hPDI** | 51 (46, 55) | 49 (45, 54) | <0.001 |
| **uPDI** | 50 (46, 55) | 51 (46, 55) | 0.037 |

## **Table S5 Associations of PDIs (in quartiles) with trajectory of CMM (specific FCMD).**

|  | PDI^a^ | | | hPDI^b^ | | | uPDI^c^ | | |
| --- | --- | --- | --- | --- | --- | --- | --- | --- | --- |
| Transition | Q2 vs Q1 | Q3 vs Q1 | Q4 vs Q1 | Q2 vs Q1 | Q3 vs Q1 | Q4 vs Q1 | Q2 vs Q1 | Q3 vs Q1 | Q4 vs Q1 |
| Baseline → Diabetes | **0.89 (0.80, 0.99)** | **0.89 (0.80, 0.99)** | **0.85 (0.76, 0.96)** | **0.80 (0.72, 0.88)** | **0.74 (0.66, 0.83)** | **0.64 (0.57, 0.73)** | **1.15 (1.03, 1.28)** | 1.05 (0.93, 1.17) | **1.23 (1.10, 1.39)** |
| Baseline → IHD | 0.98 (0.91, 1.06) | 0.95 (0.88, 1.03) | **0.87 (0.80, 0.95)** | 0.94 (0.87, 1.01) | 0.93 (0.86, 1.01) | 0.92 (0.84, 1.00) | 0.98 (0.91, 1.06) | 1.03 (0.95, 1.11) | **1.08 (1.00, 1.18)** |
| Baseline → Stroke | 0.91 (0.81, 1.02) | 0.95 (0.84, 1.07) | 0.97 (0.85, 1.10) | 0.94 (0.84, 1.06) | 1.02 (0.90, 1.15) | 0.99 (0.87, 1.13) | 1.07 (0.95, 1.20) | 1.11 (0.98, 1.25) | **1.18 (1.03, 1.34)** |
| Baseline → CMM | **0.59 (0.41, 0.85)** | **0.68 (0.48, 0.97)** | **0.62 (0.42, 0.91)** | 0.78 (0.55, 1.10) | **0.67 (0.46, 0.98)** | 0.78 (0.52, 1.16) | 0.87 (0.59, 1.28) | **1.43 (1.00, 2.04)** | 1.19 (0.79, 1.80) |
| Diabetes → CMM | 1.21 (0.86, 1.69) | 1.31 (0.94, 1.84) | 0.94 (0.63, 1.39) | 1.23 (0.89, 1.71) | **1.41 (1.01, 1.97)** | 1.21 (0.81, 1.80) | 0.90 (0.64, 1.26) | 0.99 (0.70, 1.41) | 1.01 (0.70, 1.45) |
| IHD → CMM | 0.89 (0.69, 1.15) | 0.85 (0.65, 1.10) | **0.72 (0.54, 0.98)** | 0.87 (0.68, 1.11) | **0.72 (0.55, 0.95)** | 0.79 (0.58, 1.06) | **1.30 (1.00, 1.69)** | 1.16 (0.88, 1.53) | 1.24 (0.92, 1.67) |
| Stroke → CMM | 0.84 (0.57, 1.25) | 0.76 (0.50, 1.15) | 0.73 (0.47, 1.13) | 0.93 (0.64, 1.37) | 0.73 (0.48, 1.11) | 0.75 (0.47, 1.19) | 0.90 (0.59, 1.38) | 1.35 (0.89, 2.04) | 1.23 (0.78, 1.93) |
| Baseline → Death | 0.93 (0.84, 1.03) | 0.92 (0.82, 1.02) | 0.90 (0.81, 1.01) | 0.91 (0.83, 1.01) | **0.86 (0.78, 0.96)** | **0.88 (0.78, 0.98)** | **1.13 (1.02, 1.25)** | 1.10 (0.99, 1.22) | **1.26 (1.12, 1.41)** |
| Diabetes → Death | 0.92 (0.64, 1.32) | 1.06 (0.74, 1.51) | 0.91 (0.62, 1.35) | 1.10 (0.79, 1.54) | 0.98 (0.68, 1.42) | 0.97 (0.64, 1.47) | 0.79 (0.55, 1.13) | 0.93 (0.64, 1.34) | 1.01 (0.69, 1.48) |
| IHD → Death | 0.88 (0.71, 1.10) | **0.73 (0.57, 0.92)** | 0.84 (0.65, 1.07) | 0.94 (0.76, 1.17) | 0.82 (0.65, 1.04) | 0.84 (0.65, 1.10) | 1.09 (0.87, 1.37) | 1.07 (0.84, 1.35) | 1.06 (0.82, 1.37) |
| Stroke → Death | 0.93 (0.72, 1.21) | 0.80 (0.61, 1.04) | 0.93 (0.71, 1.22) | 1.18 (0.91, 1.54) | 0.96 (0.73, 1.27) | 1.30 (0.98, 1.71) | 0.88 (0.68, 1.13) | 1.05 (0.81, 1.36) | 0.98 (0.73, 1.31) |
| CMM → Death | 1.28 (0.92, 1.77) | 1.13 (0.80, 1.59) | 0.81 (0.53, 1.26) | 0.85 (0.61, 1.18) | 0.89 (0.63, 1.28) | 1.15 (0.79, 1.67) | 0.86 (0.60, 1.22) | 0.95 (0.66, 1.36) | 1.26 (0.87, 1.83) |

^a^PDI, plant-based diet index;

^b^hPDI, healthy plant-based diet index;

^c^uPDI, unhealthy plant-based diet index


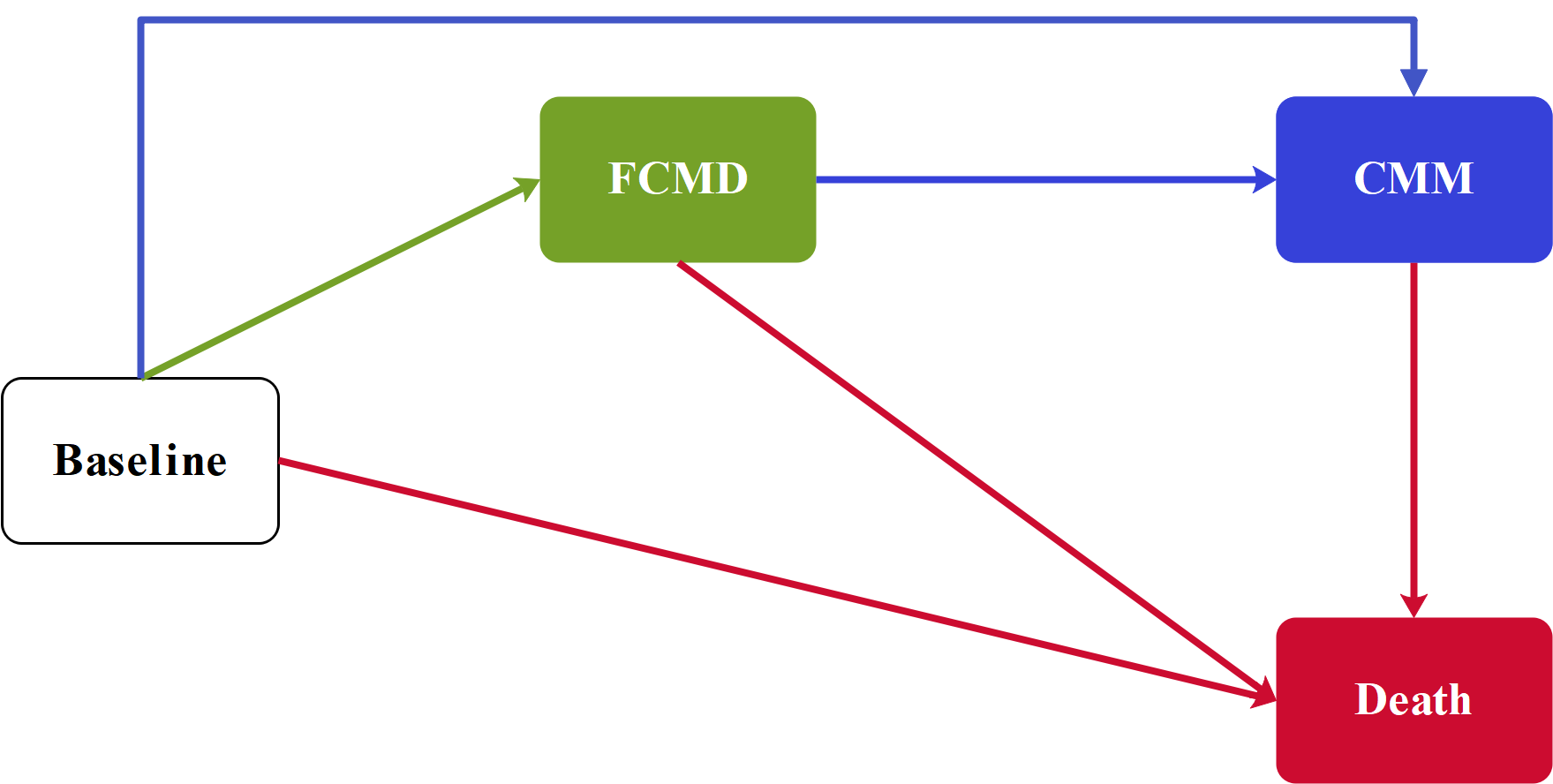


## **Figure S1 CMM transition pattern A**


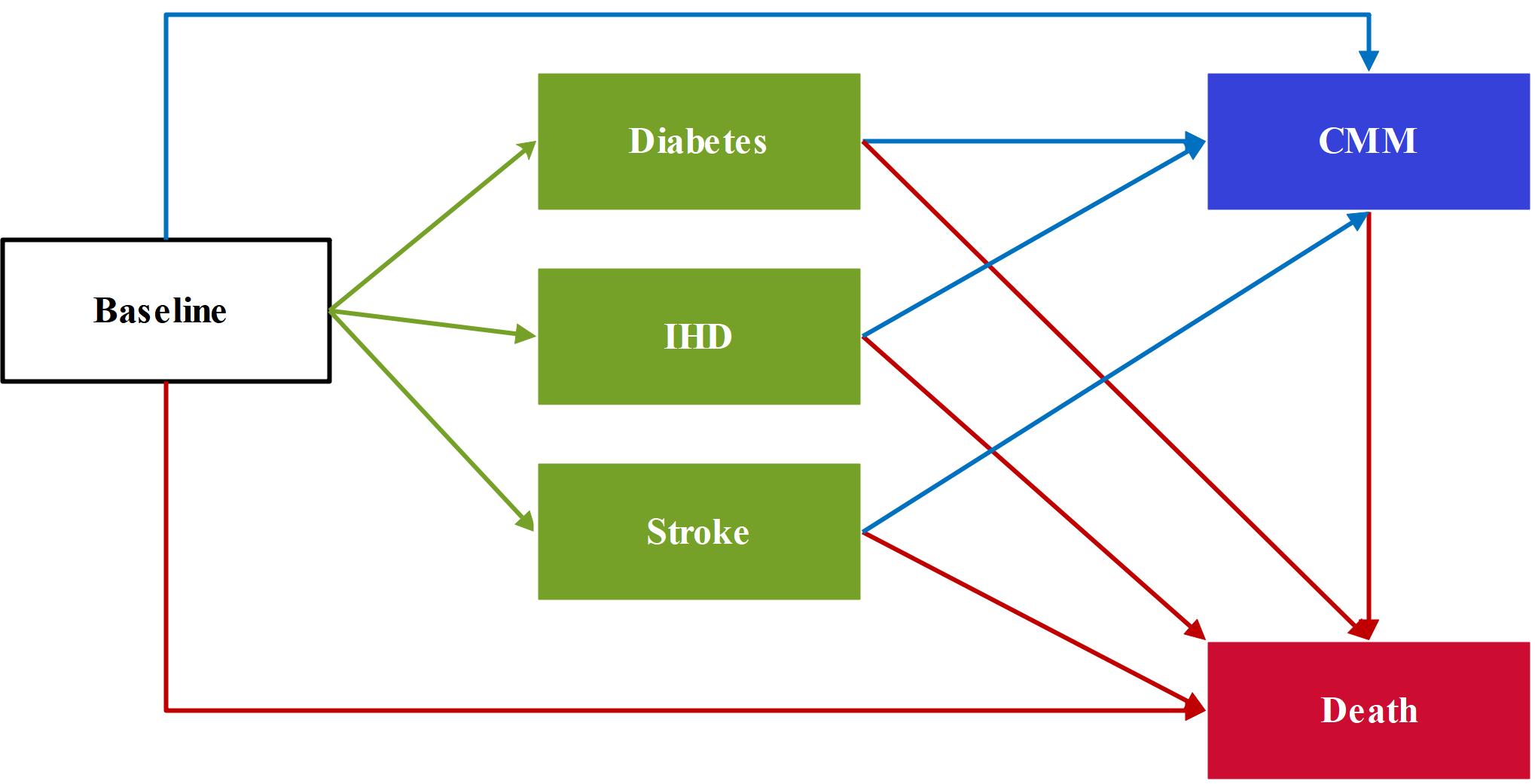


## **Figure S2 CMM transition pattern B**


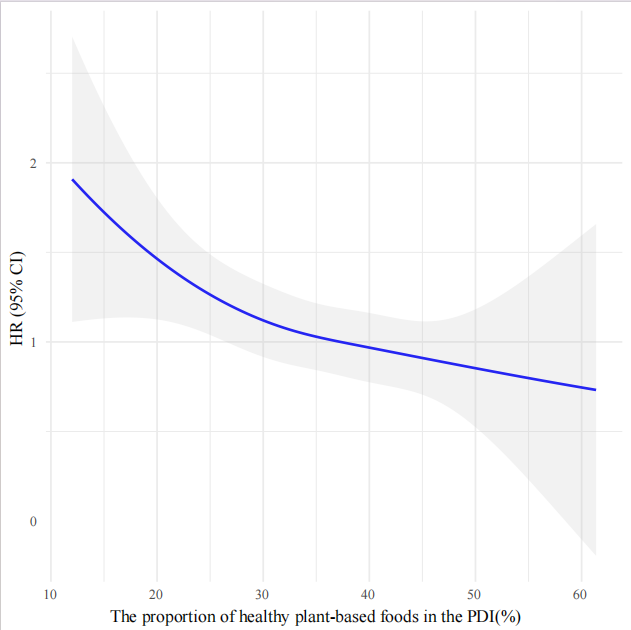


## **Figure S3 Associations of for incident CMM by proportion of healthy plant-based foods in the PDI**

**
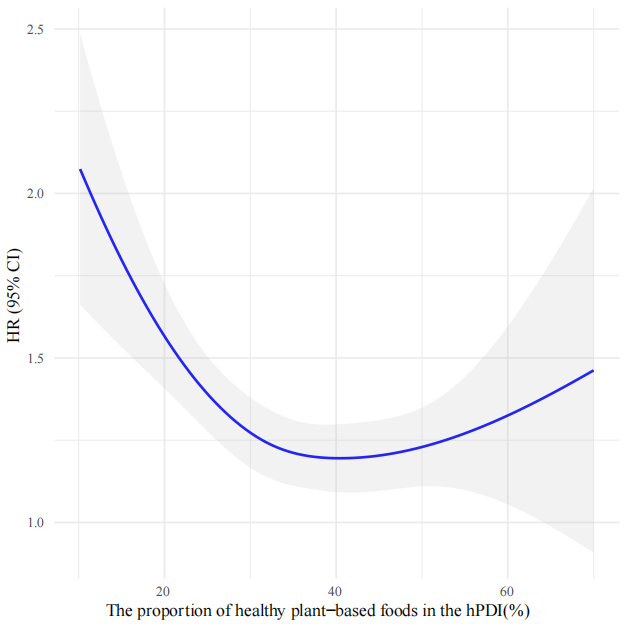
**

## **Figure S4 Associations of for incident all-cause mortality by the proportion of healthy plant-based foods in the hPDI**


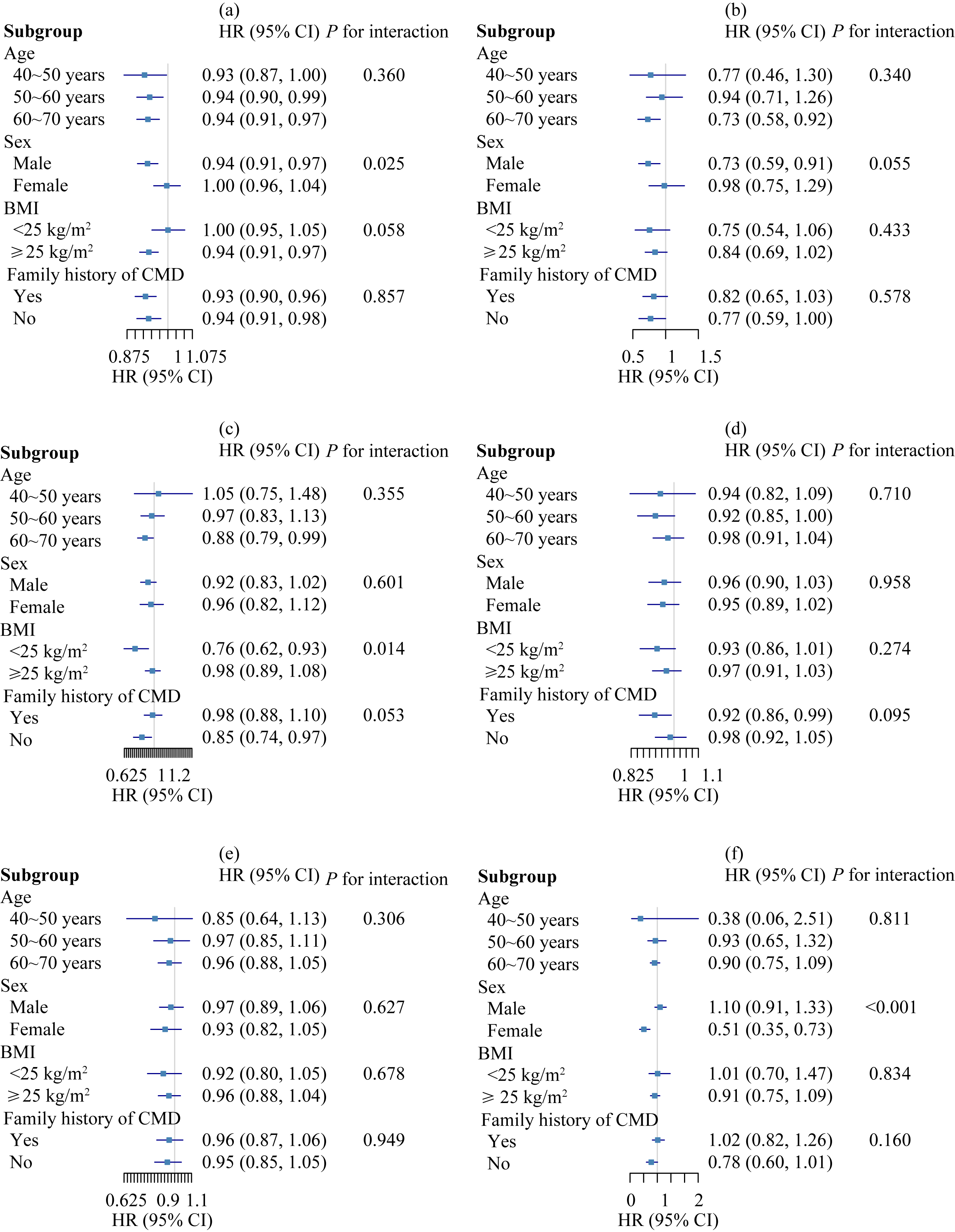


## **Figure S5 Associations of PDI with CMM transitions among 83,610 participants, stratified by potential modifiers.**

1. Associations of PDI with transition from baseline to FCMD;
2. Associations of PDI with transition from baseline to CMM;
3. Associations of PDI with transition from FCMD to CMM;
4. Associations of PDI with transition from Baseline to death;
5. Associations of PDI with transition from FCMD to death;
6. Associations of PDI with transition from CMM to death.

Abbreviation: HR, hazard ratios; CI, confidence interval; PDI, plant-based diet index; FCMD, first cardiometabolic disease; CMM, cardiometabolic multimorbidity.

Associations were expressed as HR (95% CI) per interquartile range increase in PDI.

P-value < 0.05 (Z-test) indicated significant modification.

Cardiometabolic diseases included diabetes, ischemic heart disease and stroke. CMM was defined as the occurrence of at least two of the above-mentioned diseases.


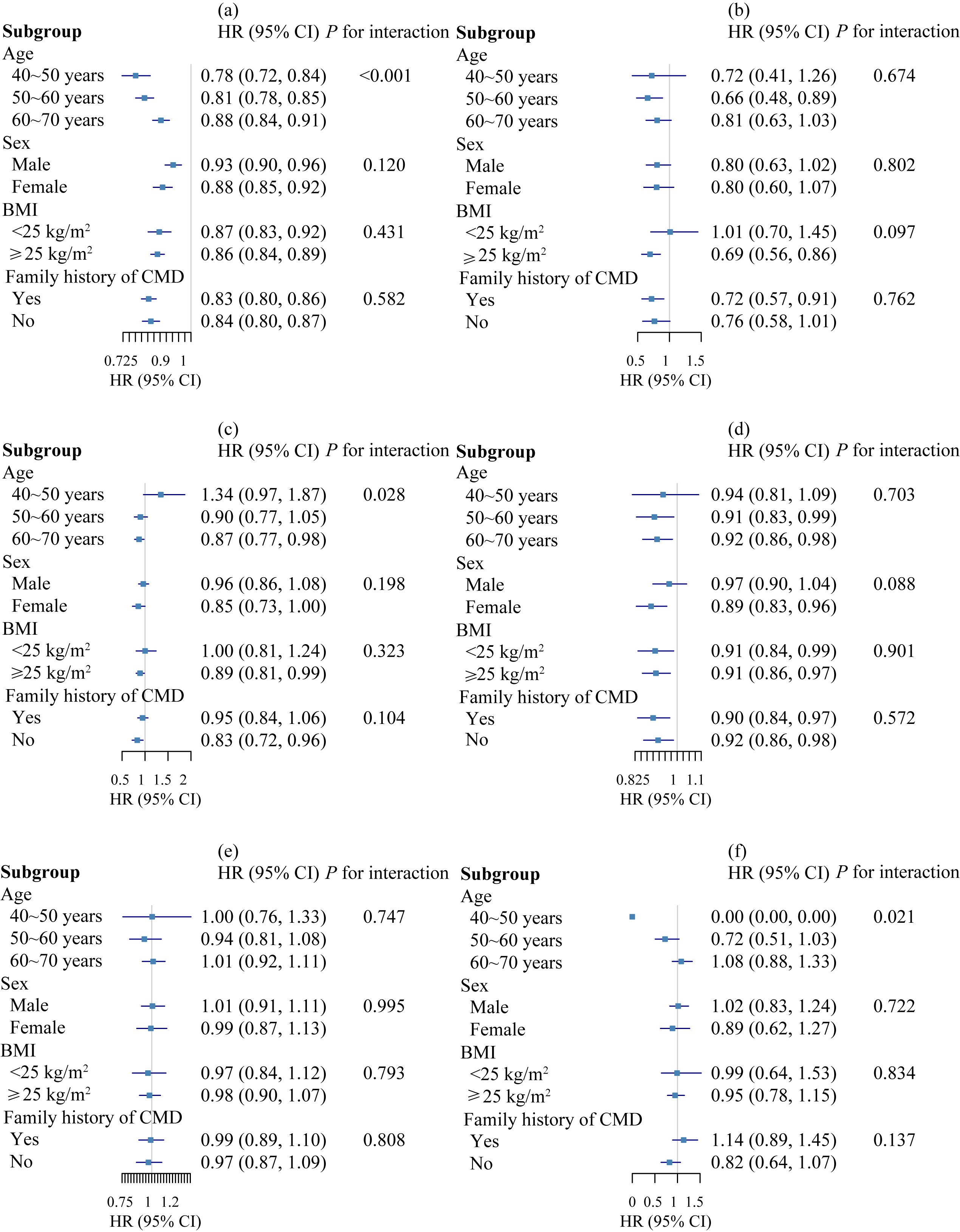


## **Figure S6 Associations of hPDI with CMM transitions among 83,610 participants, stratified by potential modifiers.**

1. Associations of hPDI with transition from baseline to FCMD;
2. Associations of hPDI with transition from baseline to CMM;
3. Associations of hPDI with transition from FCMD to CMM;
4. Associations of hPDI with transition from Baseline to death;
5. Associations of hPDI with transition from FCMD to death;
6. Associations of hPDI with transition from CMM to death.

Abbreviation: HR, hazard ratios; CI, confidence interval; hPDI, healthy plant-based diet index; FCMD, first cardiometabolic disease; CMM, cardiometabolic multimorbidity.

Associations were expressed as HR (95% CI) per interquartile range increase in hPDI.

P-value < 0.05 (Z-test) indicated significant modification.

Cardiometabolic diseases included diabetes, ischemic heart disease and stroke. CMM was defined as the occurrence of at least two of the above-mentioned diseases.


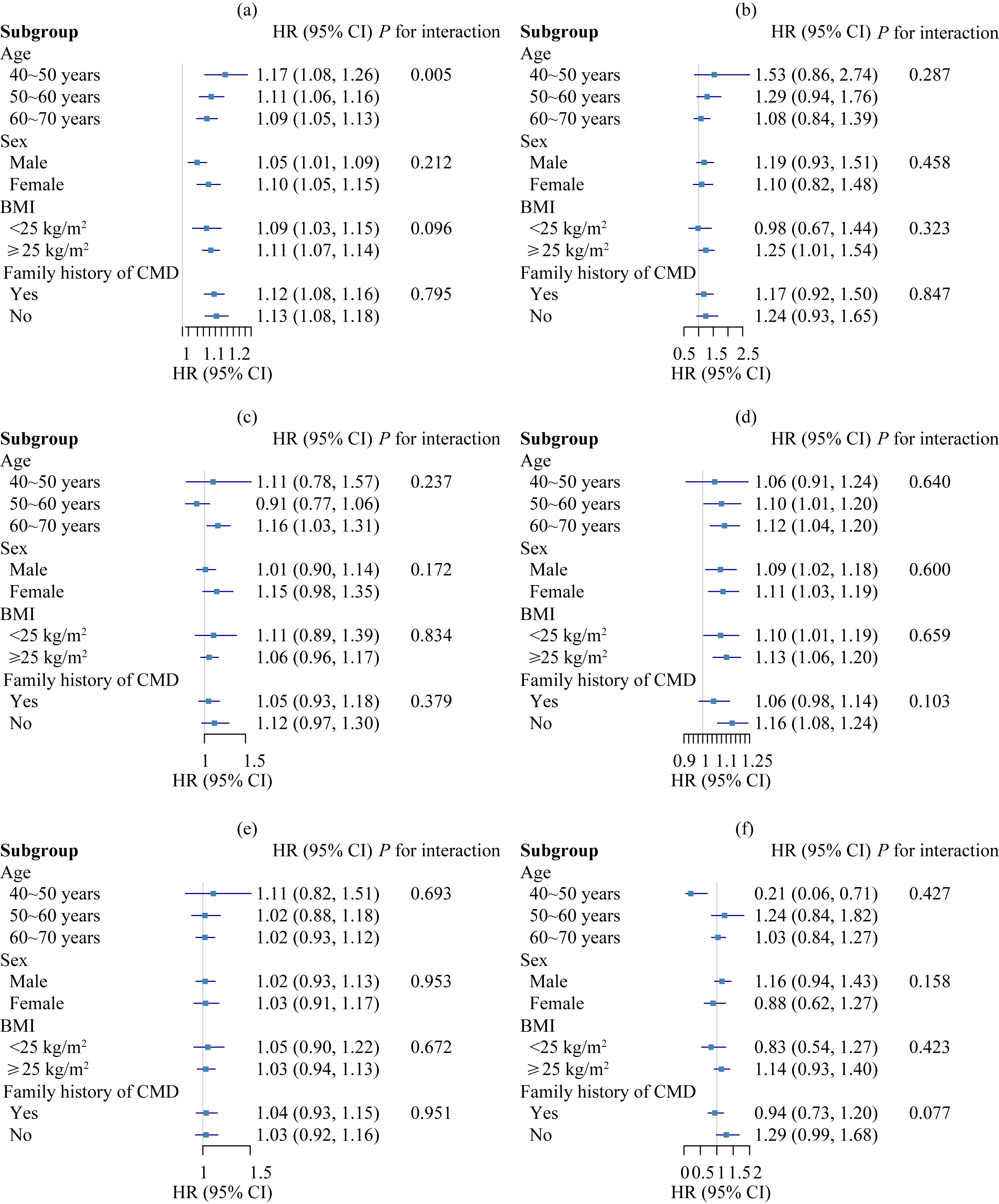


## **Figure S7 Associations of uPDI with CMM transitions among 83,610 participants, stratified by potential modifiers.**

1. Associations of uPDI with transition from baseline to FCMD;
2. Associations of uPDI with transition from baseline to CMM;
3. Associations of uPDI with transition from FCMD to CMM;
4. Associations of uPDI with transition from Baseline to death;
5. Associations of uPDI with transition from FCMD to death;
6. Associations of uPDI with transition from CMM to death.

Abbreviation: HR, hazard ratios; CI, confidence interval; uPDI, unhealthy plant-based diet index; FCMD, first cardiometabolic disease; CMM, cardiometabolic multimorbidity.

Associations were expressed as HR (95% CI) per interquartile range increase in uPDI.

P-value < 0.05 (Z-test) indicated significant modification.

Cardiometabolic diseases included diabetes, ischemic heart disease and stroke. CMM was defined as the occurrence of at least two of the above-mentioned diseases.
